# Non-Formaldehyde, Bio-Based Adhesives for Use in Wood-Based Panel Manufacturing Industry—A Review

**DOI:** 10.3390/polym13234086

**Published:** 2021-11-24

**Authors:** Diogo Gonçalves, João Moura Bordado, Ana C. Marques, Rui Galhano dos Santos

**Affiliations:** CERENA, Chemical Engineering Department, Instituto Superior Técnico, Universidade de Lisboa, Avenida Rovisco Pais, 1049-001 Lisboa, Portugal; diogo.azevedo.goncalves@ist.utl.pt (D.G.); jcbordado@ist.utl.pt (J.M.B.); ana.marques@ist.utl.pt (A.C.M.)

**Keywords:** adhesives, non-formaldehyde, review, wood-based panels

## Abstract

There is a strong need to develop and implement appropriate alternatives to replace formaldehyde-based adhesive systems, such as phenol–formaldehyde, in the industry of wood-based panels (WBPs). This is due to the toxicity and volatility of formaldehyde and restrictions on its use associated with some formaldehyde-based adhesives. Additionally, the current pressure to reduce the dependence on polymeric materials, including adhesives, from petrochemical-based sources has led to increased interest in bio-based adhesives, which, in some cases, already provide acceptable properties to the end-product. Among the potential raw materials for good-quality, renewable-based adhesive formulations, this paper highlights tannins, lignin, and protein sources. However, regarding renewable sources, specific features must be considered, such as their lower reactivity than certain petrochemical-based sources and, therefore, higher production costs, resource availability issues, and the need for toxicological investigations on alternative systems, to compare them to conventional systems. As a result, further research is highly encouraged to develop viable formaldehyde-free adhesive systems based on renewable sources, either at the technical or economical level. Moreover, herein, we also showcase the present market of WBPs, highlighting the obstacles that the alternative and new bio-based adhesives must overcome.

## 1. Introduction

As a result of new technologies, living standards across the globe have increased, with a corresponding increase in the demand for new production methods and feedstocks to sustain this growth. An industry that has been forced to adapt to address the challenge of sustainability is the wood furniture industry, which has focused majorly on the development of artificial wood panels made mostly of processed wood and an adhesive or resin [1,2]. Some artificial wood panels can also be used in other industries, such as the construction industry [3].

The global market of adhesives accounted for more than 14.7 million tonnes in 2019 [4], with the Asia Pacific region accounting for approximately 38% of the global sales, followed by North America and Western Europe. The construction sector represented more than 26% of the global demand in 2019 [4].

With the global increase in the demand for wood-based panels (WBPs), the wood adhesive industry has also grown in terms of production capacity. The typical adhesives used in the production of wood panels are mainly petrochemical-based thermosets, such as phenol–formaldehyde (PF) resins, urea–formaldehyde (UF) and melamine–urea–formaldehyde (MUF) resins, and polymeric methylene diphenyl diisocyanate (pMDI) adhesive systems. These are examples of conventional adhesive systems.

The use of these types of resins, although still being the most widely employed in WBPs due to their economic advantages and good properties, presents severe drawbacks, such as the increasing awareness of human health complications as a result of toxic volatile compound emission, namely formaldehyde and phenol [5,6], and their dependence on petroleum as well.

Most formaldehyde emissions occur during the panel manufacture process due to the formaldehyde present in the adhesive formulation. Some of the emissions are known to also occur from the wood material itself. The commonly used methods to reduce formaldehyde emissions from wood panels decrease the amount of free formaldehyde present in the adhesive formulation by incorporating additives that act as formaldehyde scavengers, such as urea and ammonia, or by adding some natural compounds such as tannins or wood bark [7].

Therefore, the employment of sustainable feedstocks in the wood adhesive production lines, while reducing the amount of dangerous, volatile compounds emitted, are among the main goals of such research lines. The new generation of wood adhesive products must maintain the physical and mechanical characteristics of the currently used ones.

Bio-based adhesives have been used for millennia [8]. However, issues related to adhesive stability in humid and wet environments were often found in their traditional use. Such characteristic poor stability may compromise the panel’s physical and mechanical properties, reducing the mechanical strength and durability of the end-product. Improving the adhesive properties and water resistance is the main priority of research in the bio-based adhesives field.

The use of natural components, such as soy protein, lignin, and tannins, as feedstocks in adhesive production presents the advantages of providing an alternative for valuing these by-products that result, for example, from industries such as in soy-based oil production and on the exploration of lignocellulosic materials. As by-products of other industries, these materials are especially attractive as feedstocks since they usually entail lower costs of raw materials, lower carcinogenic volatile emissions, and the possibility of a sustainable exploration. For example, by-products from the pulping industry, such as the various lignin types, can be used to reduce costs due to their low cost and abundance. Lignin derivatives have been reported as additives in UF resins as extenders or in the partial replacement of phenol in PF resins [9]. Some industrial applications for these resins have been reported in the last few decades but they are still unable to compete commercially with the current standardly employed resins fully.

Additionally, it should be stressed that, in order to replace a conventional adhesive system, specific features, such as reactivity and production costs, resource availability, and toxicological investigations, should be carefully considered, including life cycle assessments (LCA) at various levels (economic, environmental, and social).

Therefore, sustainably sourced adhesives must be capable of addressing the main problems caused by petrochemical-based adhesives whilst maintaining their advantages such as ease of distribution, low costs, and being stable for the required durations and at the required conditions (such as rain, humidity, pressure, etc.).

This review surveys some of the latest and most relevant developments in bio-based adhesives. Furthermore, herein, we also showcase the present market of WBPs, highlighting the obstacles that the alternative and new bio-based adhesives must overcome. This market review will assist researchers in identifying new opportunities for developing novel and innovative bio-based adhesives.

## 2. Wood-Based Panels (WBPs)

WBPs are manufactured from wood materials combined with an adhesive and bonded at predetermined press times and temperatures. The press applies heat (if needed) and pressure to activate (chemically crosslink) the adhesive resin and bond the wood material into a solid panel that should have good mechanical and physical properties (strength, stiffness, form, dimensional stability, etc.).

**Figure 1:**
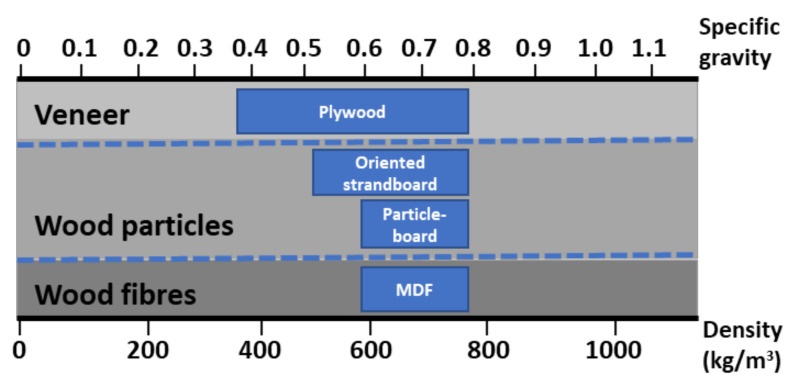
Classification of WBPs adapted from Suchsland and Woodson (1987) [10].

The most produced WBPs are particleboard (PB), medium-density fibreboard (MDF), plywood (PLW), and oriented strand board (OSB). WBPs are composite products manufactured by the effective bonding of wood materials such as fibres, particles, chips, wood powder, and veneers, among others, with various adhesives. Their usage classifies WBPs, either for structural or non-structural panels, outdoor or indoor grade panels, and the type of wood and materials used in their production. An example of this type of classification can be found in Figure 1. 

**Figure 2:**
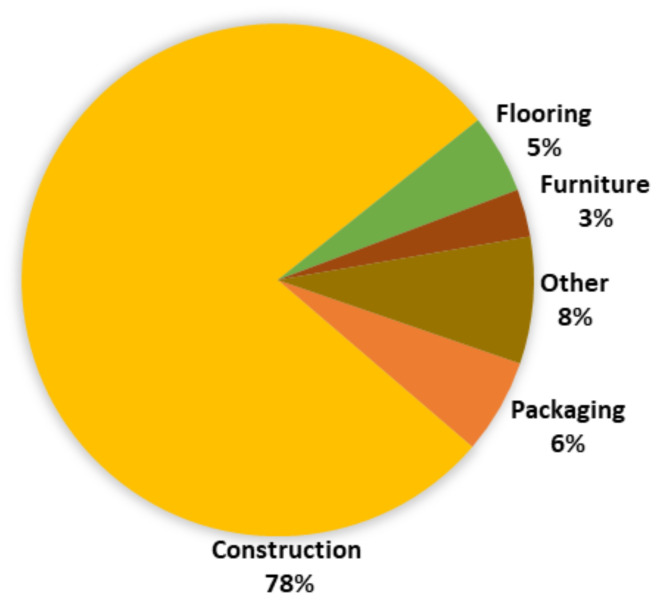
End use for OSB panels in Europe during 2019, according to [3].

Each of these products has a wide range of applications, with most of them used in the construction and furniture industries and significant use in decoration and packaging, exemplified in Figure 2 for OSB panels.

Adhesives such as the petroleum-based UF resin are the preferred adhesives in the WBP industry due to their excellent adhesion performance, even if they have comparatively lower water resistance. Meanwhile, other adhesives such as MUPF, PF, and pMDI, currently utilised by the European PB and MDF industries, amount to a negligible portion of the market, with a market share of approximately 2–3%. In the OSB industry, most of the European market uses pMDI as the primary adhesive [11].

**Figure 3:**
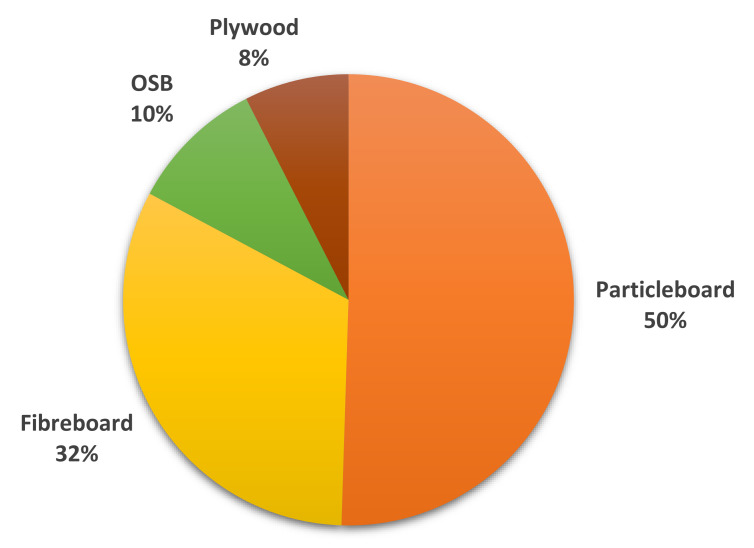
European production of WBPs during 2018, adapted from [12].

In Europe, the WBP production in 2018 was nearly 75 million m^3^, with PLW production showing the most significant drop of approximately 4.8% [12]. The WBP production in Europe was 37.8 million m^3^ for PB, 24.2 million m^3^ for fibreboard, 7.3 million m^3^ for OSB, and 5.6 million m^3^ for PLW [12], further demonstrated in Figure 3.

### 2.1. Particleboard (PB)

PB is a WBP produced by mechanically reducing the material into smaller particles, applying adhesive to the particles, and, through heat and pressure, consolidating a loose mat of the particles [13]. The particles used can be from wood chips, sawdust, waste materials, or recycled woodchips [14].

PB is typically produced in three layers; the two external ones (faces) consist of finer particles and sawdust, while the core layer consists mainly of coarser material. This way, producing a panel produces a smoother surface, which is better for laminating, overlaying, painting, or veneering [13].

Reducing lignocellulosic material to particles is less costly than reducing it to fibres in terms of materials and process. However, the resulting PB is not as strong as fibreboard due to the fibrous nature of lignocellulosic material not being exploited as efficiently [13].

**Figure 4:**
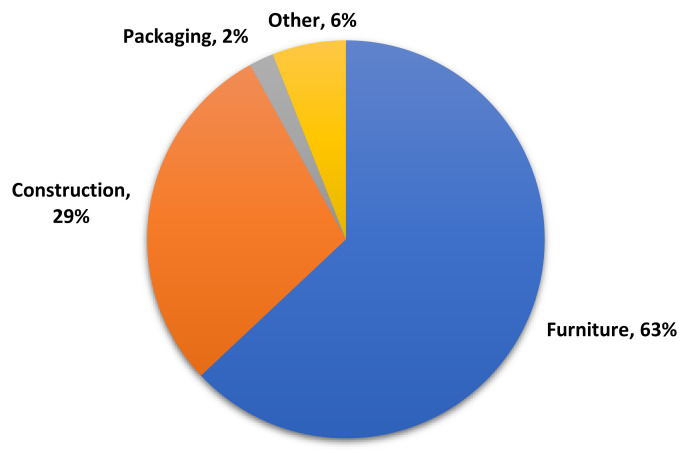
European uses for PB during 2019, adapted from [15].

PB is mainly used in furniture cores, where, due to its relatively smooth faces, other materials could be applied on top of it for decorative purposes. The end use for PB in Europe during 2019 is shown in Figure 4 [15].

Producing PB has five main steps: furnish preparation, resin application, mat formation, hot pressing, and finishing [14]. The furnish preparation requires refining the raw materials into small particles and drying them to achieve the desired moisture content of usually around 2 to 7% for most common liquid resins in use nowadays [13,14]. Developing new adhesives for use in PB manufacturing could alter the required moisture content for the particles used. In some cases, an adhesive that withstands higher moisture content could reduce the amount of energy needed for the particle drying process.

The type of resin used in PB depends on the characteristics desired, but the most used is UF resin. Based on the resin dry solid content and particle dry weight, the resin to wood ratio is usually 6 to 9% [13]. Additives such as a wax emulsion that improves water resistance could also be added in this step. PMDI adhesives can also be used in PB production to reduce formaldehyde emissions, and boards bonded exclusively by pure pMDI are marketed as no-added-formaldehyde (NAF) boards [11]. PMDI-bonded PB production has some disadvantages, such as the adhesive sticking to the press belt or plate, which could lead to the formation of holes in the PB panel and slow the manufacturing time, with the requirement of periodic cleaning processes, low tack of the mat, and a comparatively very high cost of adhesive. The relatively high base price for the adhesive can be somewhat mitigated by the manufacturing process requirement of a lesser amount of adhesive and the possibility of increasing the moisture content of the wood particles used to form the mat, which reduces the energy expenses in the initial drying process. The market for pMDI-bonded PB is still relatively small, amounting to less than 1% of the European market. Nonetheless, the advantage of producing “no-added-formaldehyde” boards makes this a promising method to invest in and explore [11]. UF adhesives are being used in the surface layer to prevent press-sticking problems to reduce some of the issues encountered in the panel manufacturing process with pMDI.

The types of adhesives used for manufacturing PB in the European market are mainly UF (90–92%), MUF (6–7%), and pMDI (1–2%), according to specific reports with data from 2016 [11].

The mat formation step requires that, after mechanically mixing the particles and the adhesive, the material goes through a continuous mat-forming system, where the material will be layered and hot-pressed at pressures between 2 and 3 MPa and temperatures between 140 °C and 220 °C. After the press cycle is complete, the panel is transported to a board cooler, where it will be hot-stacked until being sawed into finished panel sizes and sanded [14]. In the trimming process, the panel usually loses from 0.5 to 8% of its size, depending on the original size and process employed.

The principal producers of PB in Europe are Austria, France, Germany, Italy, and Poland, whilst, worldwide, the significant producers are China, Russia, Turkey, and the United States. The European Committee for Standardisation (CEN) adopted technical standards for the different types of PBs produced in Europe (EN312).

In Europe, the PB industry produces several types of WBPs according to the EN 312 standard [16], as shown in Table 1.

### 2.2. Oriented Strand Board (OSB)

Oriented strand board, commonly known as OSB, is a structural building material used for residential and commercial construction. It is a multi-layered board mainly made from strands of wood bonded with a waterproof binder under heat and pressure conditions [14].

In 2019, the primary end use for OSB was construction [3], as shown in Figure 2.

All OSB produced in Europe is classified according to EN 300 regarding its mechanical performance and relative moisture in the four grades indicated in Table 2.

**Figure 5:**
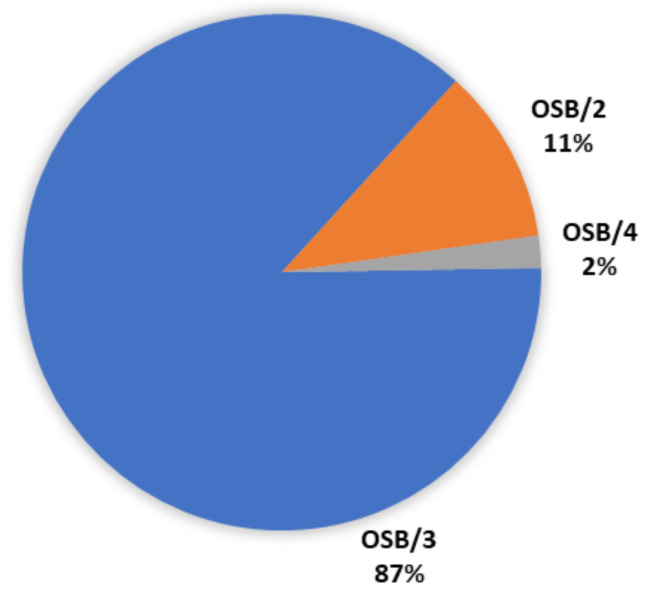
OSB categories produced in Europe during 2018, according to [12].

The production of several grades of OSB in Europe in 2019 is shown in Figure 5.

In the external layer, the strands are aligned parallel to the board length or width. On the other hand, the strands in the internal layer/layers are randomly distributed with different orientations and alignment, generally at right angles to the strands in the external layers [14]. The orientation of wood strands with a typical aspect ratio, with the strand’s length divided by its width, of 3 can produce panels with greater bending strength and stiffness in the oriented or aligned direction [13].

The manufacturing process of OSB is similar to that of PB. Typically, OSB is made from aspen poplar, pine, or other mixed hardwood and softwood logs. The most commonly used adhesives in OSB manufacturing are PF resin and pMDI. However, other resins such as MUF resins are also used to decrease the adhesive price, lowering the manufacturing expenses.

In Europe, OSB production plants use pMDI as their primary adhesive system, while North American production lines prefer PF adhesives. Manufacturing lines using pMDI usually do not require hardeners in their process, but other additives, such as special emulsifiers, are essential for better distribution of the adhesive. Other additives used could be polyols, which could accelerate the hardening reaction time, which will lead to shorter press times [11].

### 2.3. Medium-Density Fibreboard (MDF)

MDF or medium-density fibreboard panels consist of lignocellulosic fibres manufactured by the “dry process”, similar to PB production, in which resin, typically UF or PF, and other additives can be applied to the fibres. Afterwards, the adhesive-coated fibres are air-laid into a mat for subsequent pressing. Typically, there is a pre-pressing process in a band press to densify the mat. The pressing occurs at temperatures of around 140 to 165 °C for boards bonded with UF adhesives and 190 °C for PF-bonded boards [13]. The MDF boards have average densities of 700 to 800 kg/m^3^, with a core density between 600 and 700 kg/m^3^ and a face density of 1000 to 1100 kg/m^3^ [17]. Some requirements for the “dry process” include having a fibre moisture content less than 20% at the forming stage [14]. Fibres are usually obtained from a thermomechanical pulping process, which combines heat and mechanical energy to break the bonds between wood cells. In specific products requiring moisture resistance and fire retardancy, MUF resins and pMDI can be used.

**Figure 6:**
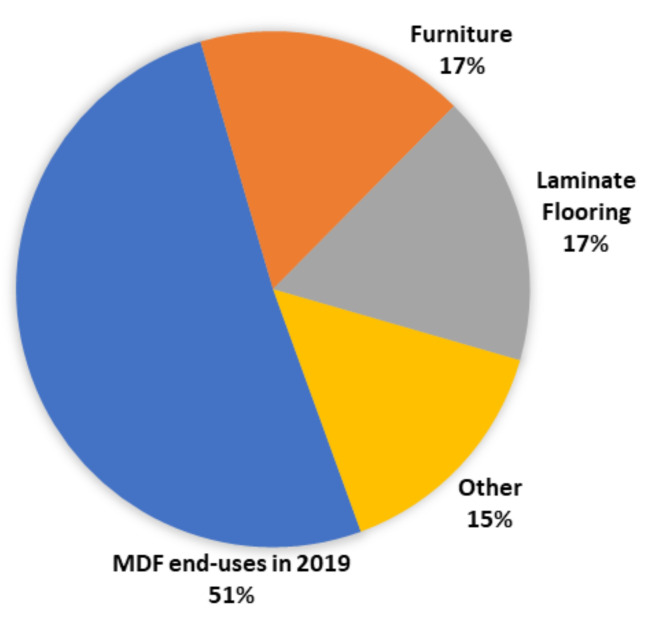
MDF panels’ end use in Europe during 2018, according to [17].

The furniture industry dominated the market for MDF products in Europe, as shown in Figure 6. Europe had a production capacity of approximately 15.1 million m^3^ in 2018, dominated by the “dry process” [12].

The different types of MDF panels produced in Europe can be found in the following Table 3.

### 2.4. Plywood (PLW)

PLW is a composite panel made from thin layers of wood veneer and a bonding agent. The layers are glued together under heat and pressure conditions. PLW can be made from either softwoods or hardwoods, and it is always constructed with an odd number of layers and with the grain direction of adjacent layers oriented perpendicular to one another [13].

The outside plies, the individual sheets of veneer in a panel, are usually either faces or face and back plies; the inner plies are the cores or centres. The core may be veneer, lumber, or PB, with the total panel thickness typically between 1.6 mm on the lower end and 76 mm on the upper end. The plies may vary in number, thickness, species, and grade of wood. To distinguish the number of plies from the number of layers, which means the changes in the grain orientation, panels can be somehow described as three-ply, three-layer or four-ply, three-layer. Generally, the outer and odd-numbered layers have their grain direction oriented parallel to the long dimension of the panel, usually the length. The grain of even-numbered layers (cores) is perpendicular to the length of the panel. The cross-layers give PLW good stability and high resistance to impacts and weather [18].

PLW is considered a material of choice in the building industry because of its outstanding structural performance, as defined by a high strength-to-weight ratio, excellent dimensional stability, and durability compared to other building materials. When compared with solid wood, PLW has properties along the length of the panel that are equal to the properties along the width; PLW panels also present superior resistance to splitting, and the form allows applications where large sheets are desirable. The use of PLW may result in more efficient use of the wood since it uses a minimum amount of wood to cover large areas while permitting the use of thinner panels than sawn lumber for some applications [13].

The PLW manufacturing process has three main stages; the first is the log preparation, the second is the veneer plain slicing or rotary cutting, drying, and grading. Finally, the third stage consists of the board lay-up, pressing, and finishing [14].

Usually, UF resins produce interior boards for dry conditions, class 1, as mentioned by EN 314-2 [18,19]. MUF is used for class 2, according to EN314-2, for use in humid interiors. PF or MUF resins are used in manufacturing, defined by EN314-2 as class 3 PLW for exterior use [18]. PLW panels are also classified according to their formaldehyde release, by the EN 636 standard, with class E1 attributed to panels with emissions of less than 0.1 ppm according to EN 717-1, and class E2 for panels with emissions above 0.1 ppm according to EN 717-1 [18].

The European standard EN 636 [1] further classifies the manufactured PLW panels according to their use, and class EN 636-1 is for PLW for use in dry conditions, class EN 636-2 for PLW used in humid conditions, which can include protected external applications, whilst also being capable of resisting exposure to the weather for short periods. This standard also applies to PLW for use in interior applications where humidity rises above expected dry use conditions. Finally, class EN 636-3 is reserved for PLW with expected uses in exterior conditions, including liquid water or water vapour in damp but ventilated locations.

**Figure 7:**
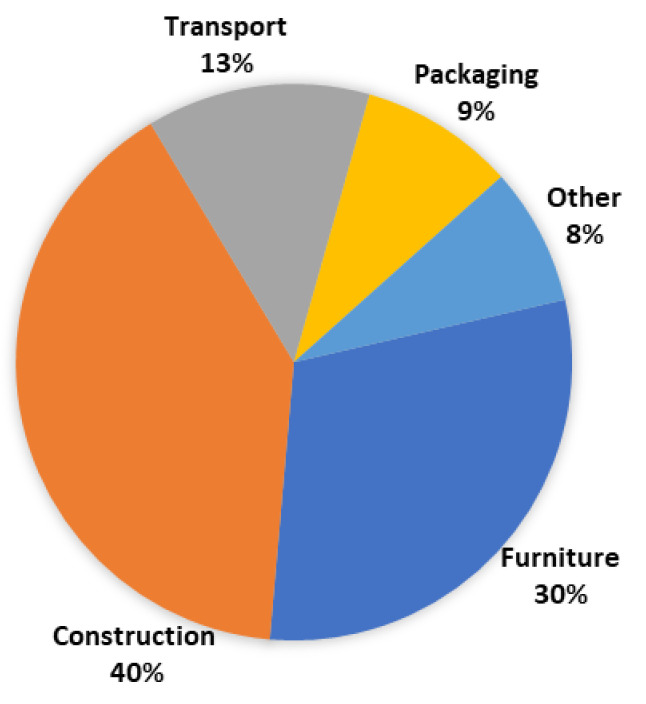
End uses of PLW panels in Europe during 2018, according to [18].

Figure 7 showcases the most common end uses for PLW in Europe during 2018.

The use of traditional adhesives, mainly formaldehyde-based ones, is favoured in the WBP manufacturing industry due to their relative low curing temperatures, excellent adhesion properties, excellent flexibility of application, low cost, and water resistance. However, they entail some significant drawbacks, such as the possibility of the release of volatile organic compounds (VOCs) and formaldehyde vapours, which pose a danger to human health, being known carcinogenic compounds [6], as well as causing other chronic illnesses. As a result, there is currently a worldwide effort by research centres and private companies to research and develop a more environmentally and human health-friendly alternative adhesive. This alternative must be economically competitive with current industry standards and provide similar mechanical and physical properties. In this regard, some biomass sources, such as soy, tannins, organosolv/kraft lignin, and cottonseed meal, among others, have been studied as feedstocks to produce bio-based adhesives [20].

## 3. Conventional Adhesive Systems for WBPs

Adhesives can be found all around us, performing several different functions. Nonetheless, a simplified definition of an adhesive could describe any type of substance capable of holding at least two surfaces together strongly and permanently [21]. Adhesives are chosen due to their properties, such as their strength.

A major adhesive market is the packaging and construction industries, which, combined, represent 80% of the demand [21]. A significant use for adhesives can be found as binders in the wood panel industry in the construction market. This industry uses mainly synthetic petrochemical-based adhesives such as UF, PF, and MUF.

Adhesives, such as UF and MUF, both thermosetting polymers of the condensation type, are the preferred adhesive type in the wood panel industry. UF adhesives are mainly used for an expected indoor-use panel, whilst the addition of melamine lowers the adhesive’s hydrolysis susceptibility, which leads to wood panels with better water and weather resistance [21], which may expand the uses for the manufactured panels. Further details on specific types of conventional adhesive systems and crosslinkers, namely formaldehyde, are given in the following sub-sections.

### 3.1. Formaldehyde

Formaldehyde is an important chemical feedstock, which acts as a crosslinker to produce phenoplast and aminoplast thermosetting resins through the reaction with other monomers (mostly urea, but also melamine, phenol, and resorcinol). It is also considered “carcinogenic to humans” by the US-based National Toxicology Program (NTP) and the World Health Organization (WHO) agency IARC, or the International Agency for Research on Cancer [5].

These formaldehyde-based adhesives are usually used in the manufacturing processes of WBPs and flooring materials, which have been identified as some of the main sources of formaldehyde emissions inside buildings, such as offices and residences [22].

The use of formaldehyde-based adhesives, such as UF and MUF as the bonding agent in the mentioned WBPs, is also considered the primary source of domestic formaldehyde emissions, with products containing UF resin having the highest formaldehyde emission rate since the resin does not cure homogeneously. As such, it still contains a large amount of UF resin that has not been cured, which, after the hydrolysis of the cured resin, results in free formaldehyde emissions. A possible solution to decrease the formaldehyde emissions rate is to reduce its content in the resin formulation. However, this can have undesirable effects on the physical and mechanical properties of the manufactured panel. Other suggestions include the use of formaldehyde-binding substances added to the resin, such as formaldehyde-binding paraffin, increasing the concentration of urea in the formulation, propylamine, and ethylamine [22]. Some other relevant factors that may influence the emission rate of formaldehyde occurring during the manufacturing process of WBPs—for instance, PB—are the pressing temperature and time, mat adhesive and moisture content, and final board density, which leads to the possibility of optimising them to reduce to a minimum the predicted emissions.

Even with stricter regulations regarding formaldehyde emissions, adhesives that incorporate formaldehyde into their formulation, such as UF and MUF (with low free formaldehyde content), are still the most used in WBP manufacturing, such as in PB and MDF [11].

These types of adhesives, even with formaldehyde present in their formulations, often fulfil the current formaldehyde emissions requirements, demanded by regulations enforced in Europe, China, and the United States. Some WBPs that emit low formaldehyde volumes, such as the expected emissions of natural wood, can be produced with special MUF adhesives [11].

### 3.2. Phenol–Formaldehyde (PF)

Phenol–formaldehyde (PF) adhesives are usually used when manufacturing WBPs requiring good durability when exposed to exterior conditions, such as OSB, softwood PLW, and siding. These types of adhesives provide better water and weather resistance to WBPs, with the downside of needing longer press times and higher press temperatures than UF adhesives, which leads to higher energy consumption and lower productivity, and containing phenol in their composition, which is a known carcinogenic and, therefore, presents a danger to human health.

Panels manufactured using PF resins may have lowered dimensional stability because of the lower moisture content in the finished products, and the inherently dark colour of PF resins may render them unsuitable for decorative product applications such as panelling and furniture [13]. However, after proper curing, these adhesives possess permanent resistance under humid climate conditions, yielding excellent adhesion to wood and panel stability [11].

The phenolic resin market was valued at USD 12.63 billion in 2019 [23]. Estimates place the value of the phenolic resin market at approximately USD 15 billion in 2021. The largest market sector for this resin would be its application as a wood adhesive, with the growth of the construction industry sector as its primary driver. The PLW manufacturing segment holds the largest share of the wood adhesive market segment [24].

Some restraints in the phenolic resin market growth are the volatility in crude oil prices, crude oil being the main feedstock for manufacturing these resins [25].

### 3.3. Urea–Formaldehyde (UF)

Urea–formaldehyde (UF) is a synthetic resin obtained by the mixing of urea and formaldehyde. It is a non-transparent thermosetting resin that exhibits some valued properties such as flexural modulus, high tensile strength, high heat distortion temperature, scratch resistance, low water absorption, mould shrinkage, high surface hardness, and elongation at break. These resins are typically used to manufacture products where dimensional uniformity and surface smoothness are of primary concern, such as PB and MDF, consuming 68% of the world’s resin production [14]. Products manufactured with UF resins are usually designed for interior applications. In 2017, PB production accounted for 46.83% of the application segment for the UF market, of which approximately 40% was used in furniture production [26]. The global market of UF is expected to grow from USD 6.53 billion in 2017 to USD 12.51 billion by 2025 [26].

UF adhesives have some significant advantages: their capacity to cure at, according to the formulation, relatively low temperatures that range anywhere from room temperature to 150 °C, with their press times and temperatures able to be moderated accordingly. Other advantages of these types of adhesives are their economic nature compared to PF adhesives and their non-flammability. However, UF adhesives have poor water resistance and still generate formaldehyde emissions, not being, therefore, a great alternative to PF adhesives in some situations.

These resins are the most widely used adhesive for composite wood products such as WBPs, with this market alone responsible for 95% of the total consumption of UF resins [14], in which, as mentioned before, PB and MDF are responsible for the majority of the market. The usually light colour of UF adhesives makes them quite suitable for the manufacture of decorative products [13].

### 3.4. Melamine–Formaldehyde (MF)

Melamine–formaldehyde (MF) resins are mainly used as paper impregnating polymers for surfacing WBPs (PB and MDF) and decorative laminate. Since MF resins produce more water-resistant products than UF resins, these are also used as adhesives in PB, MDF, and PLW production when moisture resistance is a desired property. The typically higher price of these resins limits their uses, with preference given to cheaper PF and UF resins [14].

In order to produce exceptionally durable surface coatings, melamine–formaldehyde resins can also be used in specially formulated resin systems (i.e., alkylated, methylated, butylated, or isobutylated). The coating can be either water-based or solvent-based. These resins form efficient crosslinking systems during the coating process as they react with polyester, acrylics, and epoxies. The benefits of crosslinked melamine coatings include better colour retention, wear resistance, and scratch resistance [14].

**Figure 8:**
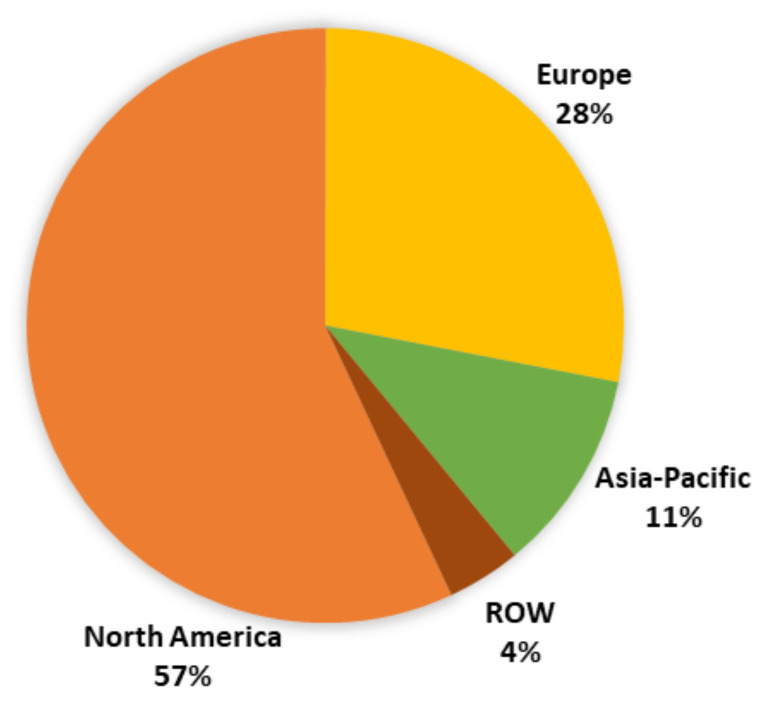
Melamine–formaldehyde markets by region, according to [27].

The MF market size it is expected to grow from USD 430 million in 2015 to approximately USD 687 million in 2022, with the largest market found in the North American region, followed by Europe, as shown in Figure 8 [27]. However, due to the COVID-19 pandemic, most predictions regarding market growth have to be revised due to the severe effects on the supply chains [28,29], such as the lack of raw materials and the transportation of products, among others.

#### Melamine–Urea–Formaldehyde (MUF)

MUF adhesives, with different proportions of melamine, are better equipped to resist moisture and environmental effects when compared with UF adhesives. Therefore, they can be used as an alternative to these adhesives in the production of PB, MDF, and, sometimes, in OSB production, when the desired properties of the final product demand it [11].

### 3.5. Methylene Diphenyl Diisocyanate (MDI)

Methylene diphenyl diisocyanates (MDIs) are used in WBP industries as an alternative to formaldehyde-based adhesives, mainly as polymeric methylene diphenyl diisocyanate (pMDI), which is primarily used in the manufacture of OSB [13]. However, it can also be used in PB [28], HDF, and MDF panels, with the resulting panels usually presenting better mechanical and physical properties.

PMDI adhesives possess high bond strength and excellent resistance to water and climate. Their higher costs are somewhat offset by their faster reaction time when compared to PF adhesives, and the lower quantity of resin required. Whilst these types of adhesives can be considered and used as formaldehyde-free in Europe, their usage in the industry requires extraordinary measures. Fully cured pMDI adhesives present no recognised health concern [11].

In Europe, the implementation of pMDI resin in WBP production is somewhat more challenging at a large scale due to the higher adhesive costs, the need for specialised equipment, and the extra safety control required during the WBP production due to the extremely high toxicity of the isocyanate particles that may be released during the manufacturing. However, once the adhesive is fully cured, it presents no further danger to human health since it will not release any more reactive particles.

Even though they can be used as an alternative for formaldehyde-based adhesives since their emissions of carcinogenic formaldehyde are null, MDI-based adhesives still provide no clear advantage when looking for a more environmentally sustainable and friendlier alternative to the former, except for requiring lower amounts of adhesive in the production of WBPs. Research is currently being conducted to develop “greener” sources for MDI production, such as the developments by COVESTRO into bio-based aniline isocyanate [30]. However, most research is still mainly performed at a lab scale.

The use of MDIs in WBP production entails special care for the water content of the wood materials since the isocyanate will react with water molecules instead of the wood components.

## 4. Testing the Adhesives

In order to assess the final properties of the developed adhesive, it is necessary to test both the adhesive and the sample of WBPs manufactured with them. The adhesive tests are used for several reasons, including the comparison of physical properties, such as tensile, shear, and peel strength, durability, and environmental resistance, among others; quality checks for a “batch” of manufactured adhesives to determine whether the adhesives are up to standards; checking the effectiveness of surface and other preparations and for the determination of some parameters that can be useful in predicting the performance of the final WBPs (cure conditions, drying conditions, etc.) [29].

Tests performed on the adhesives are essential since these tests may evaluate not only the inherent strength of the adhesive but also the optimal bonding technique, required surface cleanliness, effectiveness of surface treatments, application and coverage of the adhesive, and their curing cycle.

The tensile tests are among the most used for evaluating adhesives, with the advantage that they yield fundamental and relatively uncomplicated tensile strain, modulus, and strength data. ASTM D897 is a test method that covers the determination of the comparative tensile properties of the adhesive bonds when tested on standard shape specimens and under defined conditions of pre-treatment, temperature, and testing machine speed [31].

Some other relevant tests performed on adhesives are mentioned in Table 4.

Some testing can also be performed regarding the produced WBPs. This testing can focus on a multitude of parameters. One such parameter is evaluating the wood adhesive bond, tested according to ASTM D905-08 [38] or ASTM D2559-12a [41]. In order to interpret the measured bonding strength of wood adhesives from the previous tests, it is essential to know where the failure occurred whilst performing the mechanical tests.

There are mainly four types of failure modes acknowledged for adhesively bonded wood composites:

1. Cohesive failure of the adhesive;
2. Adhesive failure at the interface;
3. Mixed failure—a combination of 1 and 2;
4. Wood cohesive failure or wood failure.

Cohesive failure of the adhesive occurs when the failure is observed in the adhesive layers, which indicates weak bonding between the wood and the adhesive substrate, which is not desired by the WBP industry. For the second type of failure, adhesive failure, the adhesive is detached from the wood at the interface of these substrates, which implies better bonding performance of the wood adhesive. In the third type of failure, the mixed failure mode, both the cohesive failure of the adhesive and adhesive failures at the interfaces, occurs, thus showcasing better and stronger interactions between the adhesive and wood substrate, resulting in stronger bonding. Finally, in wood cohesive failure, the failure happens in the wood substrate when an entire layer of wood fibres is pulled from the respective substrate, which implies that the adhesive has penetrated the wood substrate at a depth at which mechanical interlocks and other chemical and physical interactions with the wood have occurred [42].

In WBP manufacture, the third and fourth types of failure modes are preferred since these results imply that the adhesive itself did not fail and that the panel produced is well-bonded.

Another critical parameter to test in manufactured WBPs is the water resistance of the bio-based adhesives, which can be tested based on ASTM D1151-00 [34] and ASTM D1183-03 [32]. These standard tests evaluate the produced WBPs by three main methods, each of which has its unique conditions. The first is the wet test (WT), where the bonded sample is soaked in room-temperature water, approximately 23 °C, for 48 h, and then tested immediately for wet shear strength. The second method, the water-soaking-and-drying method (WSAD), requires that the sample be soaked at room temperature for 48 h and then dried at a stable 23 °C for seven days before testing the sample’s shear strength [42], or soaked in water at room temperature for 24 h and then dried in a fume hood at room temperature for 24 h [43]. Finally, the third method is the boiling water test (BWT), and it requires that the sample be boiled in water for 2 to 4 h and then dried at approximately 63 °C for 20 h, soaked again with boiling water for 2 to 4 h, and finally cooled with room-temperature water before being tested [42,43]. The shear strength test results before and after exposing the sample to water are then compared to assess the effect of the water exposure on the sample [42].

## 5. Bio-Based Wood Adhesives

Recent regulations and industrial and societal demands have led to the renewed interest in adhesives from natural sources, also known as bio-based adhesives. These types of adhesives have been historically used in a large variety of situations, having been outclassed in their flexibility of use, physical and mechanical properties, and relative ease of manufacture by petrochemically-based adhesives. However, with the sizeable current interest in reducing industrial dependency on oil, the research into bio-based adhesives has led to new adhesive formulations with improved properties that aim to replace the industry standard.

The primary focus of research lies on abundant, relatively easy-to-produce/extract biomolecules obtained from renewable sources. The processing of these bio-based molecules can add value to materials that would otherwise be industrial waste streams. Of these, biomolecules from lignocellulosic materials have proven to be the most attractive for research since they are the most abundant and easy to valorise. However, some compounds from animal and bacterial sources are also being studied.

We will focus this review on some of the most studied family groups of biomolecules.

However, it should be stressed that bio-based adhesives have not yet shown significant importance for the European WBP industry, with their use being limited to niche products with small volumes. Nevertheless, lately, there has been an increase in the interest in bio-adhesives, mainly those derived from soy, lignin, and tannin [11]. For instance, soy-based compounds have been employed as adhesive additives, hardeners, or crosslinkers in the wood panel manufacturing industry, albeit at more minor scales than petrochemical adhesives. Some examples of soy-based adhesive systems already found in the market are the Soyad^®^ adhesive system (Solenis, Wilmington, DE, USA) and the soy protein–Kymene^®^ adhesive system (Solenis, Wilmington, DE, USA) [11].

The most representative sustainable resources for bio-based wood adhesives are described below, including some examples of their application in WBP manufacture.

### 5.1. Tannin

**Figure 9:**
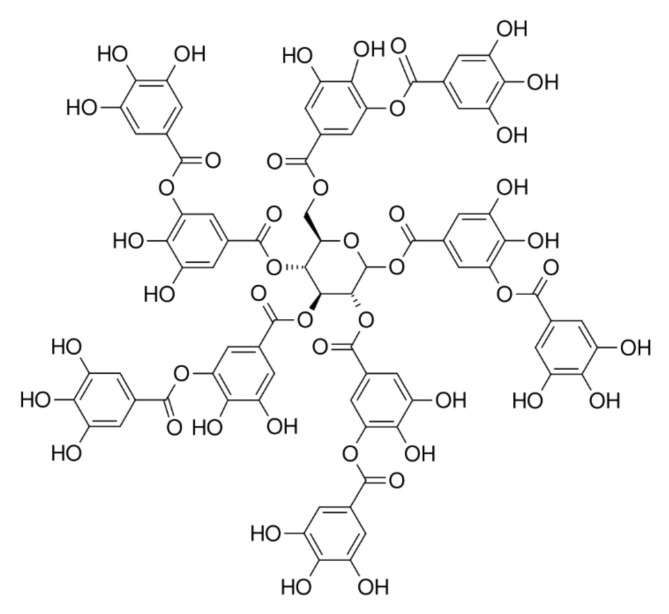
Chemical structure of tannic acid, a tannin.

Tannin can be the generic name for a substance that dissolves easily in water and whose aqueous solution is highly astringent, therefore having the property of tanning leather [44] (Figure 9). Historically, tannins have been associated with preserving hides to leather, while, chemically, the term “tannin” refers to a broad class of organic compounds.

Usually, in the adhesive industry for WBPs, only condensed tannins are utilised. The primary attractiveness of using tannins in wood adhesives is their similarity both in reactivity and crosslinking chemistry behaviour with formaldehyde, phenol, and resorcinol [45,46,47]. Condensed tannins are polyphenolic materials and generally comprise oligomeric flavonoid-type structures that are predominantly sourced from either the heartwood or bark of a variety of tree species [48].

For a long time, tannins have been commercially extracted in the southern hemisphere, mainly using bark from Mimosa Quebracho and Radiata Pine [14], with the commercial-scale extraction of other species, including hemlock, spruce, and pine species, also having been undertaken in other parts of the world.

The presence of alcohol or sugar contaminants in the final condensed tannin extract can impair the reactivity. The final extract may be composed of only 70–80% active phenolics, impacting the adhesive formulation and performance.

Tannin use in adhesive formulations requires the addition of a hardener, usually formaldehyde. Even though tannin-based adhesives with low formaldehyde emissions are commercially available, due to social and political pressures to reduce the use of formaldehyde in adhesives, non-aldehyde hardeners, such as hexamine, and auto-condensation processes have been researched, with some apparent success. Another method to reduce formaldehyde emissions is by incorporating tannins into the adhesive formulation, which was reported to reduce the formaldehyde emissions without impairing the adhesives’ mechanical performance [14].

Even though tannins are still most often used in conjunction with formaldehyde in the formulation of wood-based adhesives, other aldehydes can also be used to create crosslinking systems. The affinity of tannins toward methylol groups is the basis for the chemical coupling of tannins in PF and MUF systems with the condensation reaction of the former with the methylol groups found on phenolic or UF adhesive species as the mechanism for tannin coupling, synthesis, crosslinking, and cure, with these substrates. Outside of traditional adhesive condensation chemistries, other approaches to formulating tannin adhesives have been undertaken, such as promoting tannin auto-condensation, which reportedly produces an acceptable adhesive bond through a unique facet of condensed tannin chemistry in which the tannin oligomers are promoted to self-polymerise in order to form crosslinked polyphenolic networks. Another approach utilises the affinity of tannins for amine-based compounds to give adhesives in which the tannins are reacted into crosslinked networks on coupling polyamines [48].

It is still important to increase our understanding of the irregular reactivity of condensed tannins with aldehydes. This irregularity is primarily caused by the hydroxyl substitution patterns of different tannin extracts and has been attributed to the differences in reactivity of the phenolic rings and the different resorcinolic or phloroglucinolic tannin structures. The developments surrounding the reactivity of tannins with aldehydes, such as furfural, acetaldehyde, or propionaldehyde, as mentioned before, have been given significant attention recently, given the current interest in reducing the formaldehyde content found in adhesive formulations. Complexation with various metal ions has been demonstrated to be able to either accelerate or retard the tannins’ coupling with aldehydes [48].

Tannins have also functioned as crosslinkers in urea- and melamine-based resins to provide water resistance. Usually, incorporating tannins with UF resin requires furfural to aid in the crosslinking process. In another approach to minimise formaldehyde emissions, tannins have been combined with carbamide resins. Hybrid amino-based resins such as phenol–melamine–urea–formaldehyde (PMUF) resins have been created with tannin, providing additional crosslinking and increasing the fire resistance to the bonded product [48]. Some alternatives for tannins used in adhesive formulation without the presence of formaldehyde involve isocyanates or epoxy systems.

Tannins have been crosslinked with proteins, lignin, and starches to provide “greener” approaches to adhesive systems. Generally, the mentioned approach still uses aldehydes to couple tannins and mirror the commonly employed approaches for synthetic wood adhesive systems, using either phenolic or amino chemistry [48].

Tannin-based adhesives usually have worse physical properties, such as water resistance, when compared with petrochemical-based ones. As mentioned before, one method that tried to improve these properties used aldehydes as modifiers, as described by Zhang et al. (2019) [49], where glyoxal was successfully added to a tannin–furfural (TF)-based resin, forming a tannin–furfural–glyoxal (TFG) resin, with test results showing better dry and wet shear strengths, improving when compared with the original TF resin. These results were generally lower than those obtained by a tannin–furfural–formaldehyde-based resin (TFF), with the wet shear strength test finding of 0 MPa obtained for samples with TFG and 0.55 MPa for TFF. A sample was also tested with the addition of 12 wt% of a commercial epoxy resin to the bio-based adhesive formulation. The amount of epoxy resin added was optimised, with test results showing that the samples where 12 wt% was added achieved the overall best results. For these samples, the wet shear strength was from 0.51 to 0.58 MPa, which was not a remarkable improvement over the previously mentioned resin formulations. However, the dry shear strength improved from 1.23 to 1.82 MPa and samples of PLW produced with this modified resin presented the highest MOE measured in this study, which is even more significant than the results from samples produced using TFF resins.

For temperatures above 150 °C, samples prepared with TF and TFG resins also had higher MOE values than samples prepared via the standard PF adhesives, even though the PF resin provided the best results for wet shear strengths, 0.91 and 0.93 MPa, in the test [49].

The report by Li et al. (2019) [50] indicated that using depolymerised tannins in the formulation of a tannin–PF resin, DTPF, in which the depolymerised tannins were 40 wt% of the phenolic component (phenol comprising the remaining 60 wt%) in the resin, provided better mechanical characteristics and lower emissions than by using merely tannins, TPF, in the formulation. This study further explored the tannin-based resins by adding PEI (polyethyleneimine), as 50 wt% of tannins found in the formulation. The study results indicated that all resin formulations tested produced lower formaldehyde emissions after curing than the requirement for the manufactured panels to be considered E0 boards and presented higher bonding strength than required by the Chinese GB/T 14732-2017 [51] standard. The DTPF–PEI resin presented the lowest recorded formaldehyde emissions after curing, whilst also presenting the highest bonding strength. Contrary to what was hoped, the highest formaldehyde emissions were registered with TPF resin, as well as the lowest bonding strength. As such, even though the TPF adhesive formulation incorporates higher amounts of bio-based molecules, and, as such, reduces the dependence on petrochemical-based sources, the higher emission of formaldehyde makes this formulation undesirable since it increases the emission of the toxic component. The addition of PEI to the resin formulation increased the resin viscosity whilst lowering the respective gel times.

When using tannin-modified phenol–formaldehyde resins, it was investigated whether the mechanical properties of the produced samples improved if the tannins were depolymerised before being added to the resin, with Liu et al. (2020) [52] finding that an aqueous solution of NaOH/urea could be used for this effect. The results showcased that when the solution of depolymerised tannins was added instead of the solution of tannins, the shear strength was constantly higher, although both were consistently below the shear strength of the PF resin of 1.62 MPa.

Most studies that try to produce an adhesive using tannins whilst removing formaldehyde completely from their formulation have used lignin in its composition. Due to its attractiveness as a building block for the bio-based adhesive market and as a companion to the development of adhesives with tannins, we will summarily explore how lignin is obtained from lignocellulosic materials, as well as some of its uses in adhesive formulations primarily for WBP manufacturing.

### 5.2. Lignin

**Figure 10:**
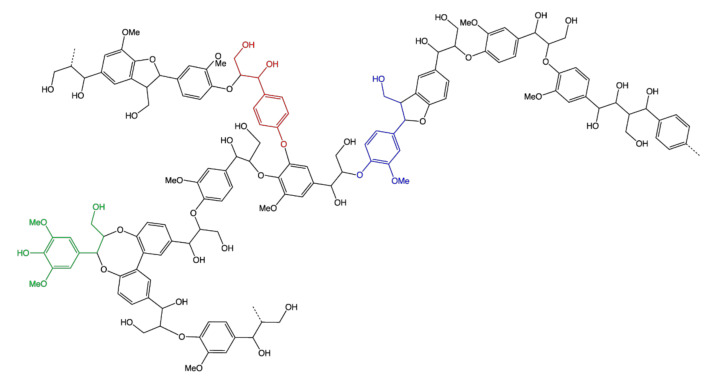
Chemical representation of a possible lignin structure and its components.

Lignin is the second most abundant biological macromolecule, usually found in lignocellulosic materials such as wood and agricultural residues, among others. Usually, it is a high-molecular-weight polymer based on aromatic phenylpropane units found in a densely crosslinked structure (Figure 10). Mixed with the other major types of polymeric chains found in the lignocellulose structure, lignin acts as the “glue” that binds cellulose and hemicellulose chains together, thus providing increased rigidity to the structure, as well as higher microbial resistance to the cell wall.

Lignin was demonstrated to be bound through covalent bonds with carbohydrates, forming lignin–carbohydrate complexes (LCC). Besides the covalent bonds, hydrogen bonding with cellulose has also been found to occur, making the LCC structure even more complex. In order to isolate lignin from wood, it is required to cleave the covalent bonds between the lignin and carbohydrates [53].

Lignin is one of the most abundant bioresources, with approximately 150 billion tons extracted annually. In the past few decades, wood-derived lignin has attracted scientific and industrial attention due to its availability and versatile properties. The global market of lignin was valued at over USD 730 million in 2018, with an expected consumption of over 1.7 million tons by 2025, up from 1.1 million tons since 2014 [54,55].

The demand for alternatives to the petrochemical industry has led to increased research and investment in several bio-alternatives. However, the significant interest in sugar-based platforms that can be used to produce biofuels has led to a slowdown in investment in other wood-based chemicals such as lignin-based chemicals. Besides being overlooked in favour of the more established sugar-based platform, another significant obstacle in developing the lignin-based chemical industry is the lack of funding options for biorefineries [53]. Lignin valorisation in the industry can be economically viable since these processes have, as a basis, a low cost and worldwide broadly available source. However, the process of lignin valorisation is not yet actively pursued at an industrial scale since there is still a need to first develop cost-effective and “greener” methodologies, infrastructures, and supply lines. The recent advances in lignin extraction processes are, therefore, an essential initial step towards the valorisation of this polymer [53]. A significant source of industrial lignin is the pulp and paper industry, which extracts approximately 50–70 million tons of lignin per year. Most of this extracted lignin is used in the form of black liquor and used as energy input in the pulping process [53].

Technical lignin is obtained as a by-product of several separation processes that most lignocellulosic materials undergo for some of their transformation processes. The composition and characteristics of technical lignin may vary according to the type and characteristics of the separation process, so, in order to assess the usefulness of the obtained lignin for use in other processes, each technical lignin batch must be considered [56].

Lignin molecular chains are consequently broken down into smaller molecules. The final product is usually identified by the commercial process by which it was obtained, such as lignosulfonates or sulphite lignin, kraft lignin, soda lignin, hydrolysis lignin, and organosolv lignin [57].

Lignin-based wood adhesives are the basis of some promising strategies for integrating biorefineries into the wood sector. As mentioned, lignin streams differ significantly in their composition and characteristics according to their extraction method and source. For example, kraft lignin contains significant amounts of sulphur, whilst organosolv lignin does not. The mentioned streams require some chemical modifications to increase their reactivity for the synthesis of bio-resins [20]. At present, kraft lignin showcases the largest range of applications with middle- and high-value products, with steady availability of its source. The prices of lignin vary widely according to final product purity and production costs. Even though lignin can be used as a biomolecule to replace phenol in PF resins, depolymerised kraft lignin obtained from biorefineries is not yet price-competitive when compared to phenol. Studies reveal that up to 70% of phenol in lignin–phenol–formaldehyde resins used in PLW manufacturing could be replaced by lignosulphates whilst maintaining the PLW boards’ characteristics. As an alternative to formaldehyde, other aldehydes such as glyoxal have been used to produce adhesives prepared and used in the WBP manufacturing industry [53].

#### 5.2.1. Kraft Lignin

The kraft process implies mixing alkaline chemicals such as NaOH and sodium sulphide (Na_2_S) with lignocellulosic materials and then introducing high temperatures, from 140 to 170 °C, obtaining what is commonly referred to as black liquor, containing degraded lignin, oxidised inorganic compounds, and other organic materials. In order to separate the kraft lignin from the black liquor, it is required to undergo an acidification process [56]. The resulting lignin may have impurities such as sulphur, which must be carefully considered when assessing its potential uses.

Kraft lignin is a product of the sulphate pulping process, with an estimated global production of 55 to 90 million tons, of which only around 2% is commercially used for value-added products [53]. Even though the kraft process is the most predominant pulping process, the recovery of kraft lignin chemicals is a market that has not been fully explored, with plenty of opportunities to grow through investment and research.

#### 5.2.2. Sulphite Lignin

Sulphite lignin is the most commercially available lignin produced in the sulphite pulping process, which uses calcium or other (bi)sulphites. The lignins obtained through the sulphonation process are water-soluble and, on average, have higher molecular weights [55]. The development of kraft lignin production has contributed to the decrease in the production of sulphite lignin from 20 million tons in the 1980s to around 7 million tons nowadays [53]. The production of sulphite lignin has decreased in western regions, such as decreases in Europe and North America, with Japan also following this trend. By contrast, countries such as India and China show increased interest in this production process [58]. The decrease in pulp production via the sulphite route has other reasons beyond the higher versatility of the kraft process, including the lower availability of ligonsulphites.

#### 5.2.3. Organosolv Lignin

Organosolv lignin is obtained by the use of organic solvents in the processing of lignocellulosic materials. This lignin extraction process preferentially cleaves the carbohydrate–lignin bonds, leading to high-molecular-weight lignin without significant chemical modifications [56]. The resulting organosolv lignin is easily recoverable and possesses features similar to native lignin, with high purity, chemical reactivity, being sulphur-free, depending on the organic solvent used, and low molecular weight. Organosolv lignin is not yet commercialised in large volumes. It still requires elevated capital investments to implement manufacturing plants, with high costs associated with the technology and formation of supply lines, which have not achieved the dimensions necessary for industrial-scale processing sites [53].

Lignin can be added to a typical PF resin formulation to increase the use of bio-based materials in resin formulations. Zhang et al. (2013) [59] reported on the use of lignin resulting from waste streams from different biorefinery processes. The study indicates that lignin can adequately replace from 30% to 50 wt%, depending on the lignin source, of the phenolic content present in the formulation of PF resin used in PLW manufacturing whilst maintaining most of the physical properties.

As mentioned, kraft lignin can be used to replace phenol in PF adhesive systems for use in WBPs, such as in PLW and OSB manufacturing. A replacement of 50 wt% was considered optimal to preserve the resin viscosity, storage stability, and bonding ability properties. The press time in the PLW manufacturing had to be increased by 30% at 150 °C, for the resin with kraft lignin added, in order to compensate for this resin’s lower curing rate compared with typical PF resin [60]. In OSB production with this resin, it was found that the addition of kraft lignin did not negatively impact the mechanical or physical properties of the final product. Some mechanical properties such as MOE and MOR were measured to be approximately 2539 and 20 N/mm^−2^, respectively, which are similar values to those obtained with a traditionally used PF resin, approximately 2400 and 22 N/mm^−2^, respectively. For the physical properties, the density, water absorption at 2 and 24 h, and thickness swelling at 2 and 24 h were, respectively, 0.65 g/cm^−3^, 49.68%, and 66.61% for 2 and 24 h, and 11.4% and 17.38% for 2 and 24 h. These results were similar to those obtained by the control sample made with PF resin, which was 0.66 g/cm^−3^, 56.08%, and 69.51% for 2 and 24 h, and 9.03% and 12.99% for 2 and 24 h for the same properties and tests. These results imply that even though there was higher water absorption in the control samples, the thickness swelling was more pronounced in the sample made with kraft lignin mixed into the adhesive [61].

Usually, lignin is not very reactive with crosslinking agents such as formaldehyde, so it has to first undergo a methylolation or hydroxymethylation step, where the reactivity of lignin molecules is improved by the introduction of reactive functional groups [62]. This methylolation step is usually carried out with formaldehyde. Due to current efforts to remove and replace formaldehyde in the production of adhesives for WBPs, alternative aldehydes such as glyoxal have been used instead in the methylolation step. Glyoxal is a non-toxic, non-volatile dialdehyde that can be used as an alternative to formaldehyde in lignin- and tannin-based wood adhesives. Even though glyoxal is less reactive than formaldehyde, it is not as toxic as the latter, which currently makes it an adequate replacement [63,64]. Studies indicate [65] that glyoxalated lignin has improved reactivity compared to original kraft lignin and that the total replacement of formaldehyde by glyoxal during the methylolation step can be done, with the produced glyoxalated lignin still being a suitable raw material for adhesive production.

Lignin has also been used as a base for adhesives in conjunction with tannins, as in the findings of Bertaud et al. (2012) [58], in which an adhesive for use in particleboard production was formulated with a composition of 60% commercial mimosa tannins with hexamine and 40% glyoxalated softwood kraft lignin. Using this adhesive formulation, the resulting particleboard had an internal bonding strength (I.B.) of 0.53 MPa, which is higher than the I.B. > 0.35 MPa required by the European standard EN314-2 [19].

A formulation for a bio-based adhesive was tested without incorporating any synthetic resin, based chiefly on lignin with a low molecular mass and tannins. The lignin with low molecular mass was obtained as a by-product of pulping wheat straw with an acid such as formic or acetic acid. Firstly, lignin was modified with glyoxal under alkaline conditions, then mixed equally with tannin, and a crosslinking agent added. In the mentioned study, hexamine was used (added as 5 wt% of tannin). The results obtained for a sample of this adhesive used in the manufacture of a WBP showed that the internal bonding strength of the bonded panel met the requirement for interior panels by the European standard EN312 [16]. In addition, the adhesive formulation, comprising lignin, tannin, and hexamine, can be considered a zero-formaldehyde-emission adhesive, based on the desiccator method test [42].

A particular example of a formulation for a lignin-based adhesive was reported by Faris et al. (2016) [43], made up of a base of a tannin solution with added hexamine, and glyoxalated lignin polyols in a 60/40 (*w/w*) solid proportion. An amount of 10 to 20% of polyethylenimine (PEI) was added to this base formulation, and the PLW samples were prepared. The adhesive formulation had lower gel times as the added PEI amounts increased up to the mentioned 20%. In the same vein, the increases in the added PEI had positive effects on the tensile strength, with its values increasing in either dry, wet, or under the effect of boiling water conditions, as the proportion of PEI also increased.

Another example of a lignin-based adhesive formulation is the mixture of glyoxalated lignin, mimosa tannins, and pMDI, 55 wt%, 25 wt%, and 20 wt%, respectively, achieving a resin with approximately 80 wt% of natural polymers [66]. The study by Lei et al. (2008) [66] showcased that a PB manufactured with the mentioned adhesive mixture possessed good internal bonding, and, through testing, it was concluded that the produced samples met the requirements for interior-grade WBPs. It was also found that during the adhesive formulation, if lignin with a lower molecular weight was used, the adhesive ended up performing better, with registered increases in the internal bonding strength. It was furthermore concluded that the introduction of triacetin, an accelerator used in aldehyde/phenolic condensation processes, into the lignin-based adhesives did not improve the properties of the resin, having even hindered the curing process. The same study showcased that an adhesive formulation comprising mainly glyoxalated lignin and pMDI at a proportion of 60/40 wt%, with resorcinol added instead of triacetin, provided good results. This formulation is not scalable to commercial manufacture due to the high costs of pMDI and resorcinol. The previously mentioned formulation also did not address the need to increase the use of biomolecules, an issue addressed by the formulation that included tannins in its mixture, with positive results in the form of the higher values of MOE reached by PB produced with this resin.

Researchers have also tested the effects of modifications in a tannin solution, used in an adhesive formulation composed of the mentioned tannin solution and glyoxalated kraft lignin with a solid proportion of 60 to 40% (*w*/*w*), respectively. In order to improve the water resistance of the final resin and diminish or eliminate the formaldehyde emissions that may occur during the resin curing step, a hydroxyl-terminated oligomeric precursor of a hyperbranched poly(amine-ester) was added to boost the internal structure of the resin, as well as furfuryl alcohol, which served as a crosslinking agent [67]. Comparing the original tannin/glyoxalated kraft lignin (TGKL) resin, the one made with the modified tannin solution (MTGKL), and a commercial phenol–formaldehyde one (CPF), it was discovered that the MTGKL resin provided the best panel characteristics, with a dry tensile strength of 39.72 MPa, an elongation at break of 21.91%, and a maximum load of 5506.9 N, followed by TGKL resin, which had a dry tensile strength of 28.79 MPa, an elongation at break of 20.61%, and a maximum load of 3720.57 N.

The CPF resin had a maximum load below 200 N, a dry tensile strength of 1.39 MPa, and elongation at break values from 12.45% to 17.3%. The two bio-based adhesives were better than the CPF resin in almost every test but the water-resistant ones. After soaking the produced samples in cold water for 24 h, the samples made with TGKL resin showed delamination, while those made with CPF and MTGKL yielded values of 1.25 and 27.62 MPa, respectively. When submitted to boiling water soaking for 2 h, the CPF samples still presented a 1.01 MPa tensile strength, while the samples made with MTGKL resin showed delamination [67]. Therefore, it was concluded that the modification of the tannin solution helped improve the water resistance of the final resin.

### 5.3. Proteins

Proteins are linear polyamides formed by amino acids linked together by polypeptide bonds and are essential building blocks found in all living organisms. There are 20 different amino acids with either acidic, basic, or neutral properties, depending on the structure of their side chain [68].

The properties of a protein can be attributed to its complex structure. The primary structure comprises the amino acid sequence, which can form a secondary structure by partly organising into α-helices and β-sheets. This tertiary structure accounts for the existence of side chains, which interact to form a 3D structure, and, finally, the quaternary structure, in which the whole protein molecule interacts with other protein molecules to form a higher order [69].

Proteins have long been used as binders for several different uses, such as wood adhesives [8]. In wood adhesives, they were eventually replaced by petrochemical-based polymers due to cost efficiency and use. Poor bond strength, water resistance, and environmental resistance were all factors that pushed the industry away from these bio-based adhesives [68].

The functional groups found in the side chains of the primary polypeptide chain are the main factors in the hydrophilic or hydrophobic behaviour of the amino acids, and these also provide possible points of interaction with hydroxyl or carboxyl groups found in wood, which may result in crosslinking [68].

Generally, protein-based adhesives suffer from high viscosity and low solid content. Usually, they also have poor water resistance, which restricts their use to indoor applications. Improving these characteristics is a significant focus of research nowadays to extend the applicability of wood-bonded protein adhesives.

Physical and chemical methods have been used to improve the properties of protein-based adhesives, one example of which is protein denaturation. The denaturation of the native protein structure works by exposing functional groups that are usually hidden within the protein’s 3D structure, which may enable better solubilisation and bonding. The increased solubilisation improves the flow of the protein-based adhesive over the wood surface, permitting the formation of hydrogen bonds with the wood surface and allowing for subsequent chemical crosslinking [68].

Protein denaturation can be triggered by increases in temperature, changes in pH, and by the addition of denaturants such as alcohol, urea or guanidine hydrochloride, borax, sodium sulphite, enzymes, sodium dodecyl sulphate (SDS), or other detergents [68,69,70].

Typical examples of sources of protein used and researched for adhesive production are the soybean and cottonseed.

The use of protein from a plant source as a feedstock in the adhesive industry is preferred to an animal-sourced one due to the lower costs and environmental impact. From the several plant sources of protein available, the selection of one must consider, beyond the properties of the final adhesive produced, the competition for the bio-resource from other industries, which could lead to higher prices or excessive production of the monoculture. This overconsumption is often preceded by deforestation, which inevitably leads to a higher environmental impact. As an example, soy protein, which is the most common source of protein in the bio-based adhesive research and industry, is also used in the food industry and as a feedstock in the animal feed industry [71]. Other types of protein sources that are also a possible cause for conflict are peas or wheat. Some less contested and therefore more desirable sources of proteins would be, for example, grass [72], algae [73], and microalgae [74].

#### 5.3.1. Soy

Soy protein is one of the primary feedstock sources and a research focus for the commercial production of bio-adhesives for use in the WBP sector as a bio-alternative to PF- and UF-based adhesives. Some advantages of using soy protein-based adhesives are their low cost, ease of handling, and low pressing temperatures, whilst some drawbacks are their poor water resistance and possible biological degradation [20].

Soy proteins mainly consist of 18 different amino acid monomers. Some side chains found in soy proteins can interact and/or react with organic or inorganic substances and cellulose fibres. These side chains can be chemically, physically, or enzymatically modified to achieve desired properties. The protein molecules can dissolve and unfold in solution. In the latter case, the increase in the surface area also increases the contact surface area. These unfolded molecules become entangled with each other during curing, which improves the bonding strength [75].

Soybean protein is readily available and can be extracted directly from soybean seeds or from soybean meal obtained after processing in an oil refinery. Soybean meal is produced at a rate of approximately 4 kg for each 1 k of soybean oil [22,76]. This protein source has been used as an ingredient for adhesives in several forms, such as enzyme-treated soy flour, oxidant-modified soy flour, and chemically denatured soy protein.

In terms of dry adhesive strength, soy protein adhesives have shown excellent adhesion performance, equivalent to that of formaldehyde-based adhesives, but have constantly presented weaker water resistance. This weak water resistance can be due to the hydrophilic groups found in the external layer of soy proteins. As such, improving the water resistance of soy protein-based adhesives is one of the main focuses of worldwide research. Approaches such as protein modifications present positive results, with some of the modifiers being urea, guanidine hydrochloride, ethylene glycol, carboxylic acids, cationic detergents [77], clay calcium carbonate, SDS, and sodium dodecylbenzene sulphonate [78], among others. The tests on adhesives with the mentioned modifiers presented, among other characteristics, improved the water resistance.

As described by Vnucec et al. (2016) [76], the main chemical modification methods for soy protein adhesives can be divided into four categories, with the first being the denaturation of soy proteins that occurs by breaking their internal structure; the second is the molecular modification of soy proteins, which focuses on grafting reactive groups of chemical reagents onto protein molecules. After passing curing processes, these groups will react with the protein’s polar groups and form a crosslinked network. The third and fourth categories both involve the mixing of soy protein products with either natural materials such as lignin and tannins or with synthetic resins, such as PF, melamine–formaldehyde (MF), MUF, and epoxy resin (EPR) [79], respectively [76].

Urea can function as a denaturation agent that unfolds the secondary, tertiary, and quaternary structures of a protein. The oxygen and hydrogen atoms present in urea molecules can actively interact with the hydroxyl groups found in soy protein, breaking down the hydrogen bonding that occurs in the protein body and, therefore, unfolding the protein complex. Soy flour has an enzyme, urease, that could increase the hydrolysis rate of urea to carbon dioxide and ammonia, weakening the effect of urea modification. Therefore, a urease inhibitor such as nBTPT, N-(n-butyl) thiophosphoric triamide, is needed, which can inhibit the urease catalyst action and consequentially enhance the adhesive strength [75].

Citric acid is an essential agent for promoting crosslinking with substrates found in polymeric chains from soy and cotton, for example, since it contains carboxyl groups that may interact with the amino groups present in soy protein. In order to promote the interaction between the citric acid and the carbohydrates found in soy, sodium hypophosphite (NaH_2_PO_2_) could be used as a catalyst [75].

As with citric acid, boric acid has also been reported to interact with carbohydrates in soy flour and create crosslinking within the carbohydrate complex, resulting in a more robust polymeric network that adds resistance to the adhesive and provides a significant decrease in water absorption in the case of soy plastics [80]. Since water absorption is a common problem for WBPs fabricated with bio-based adhesive systems, water resistance for these panels could be improved by introducing boric acid in the resin [75].

Sodium hydroxide, NaOH, can cleave internal hydrogen bonds in coiled protein molecules, so it can be used to unfold protein molecules and expose them to available polar groups for stronger adhesion [75]. A urea-treated soy flour adhesive modified with 0.30% *n*BTPT resulted in significantly higher mechanical strength. Even greater strength was noted with the addition of NaOH and 7% citric acid. Thickness swell and linear expansion were reduced at 0.40% *n*BTPT, and thickness swell was improved with the addition of 9% citric acid [75].

Modifiers such as aspartic, glutamic, or acetic acid revealed no significant improvement compared to tensile strength and elongation at break values obtained with only an SPI adhesion system [81].

Another modifier tested to improve the final properties of a soy-based adhesive was a waterborne epoxy emulsion (WEU), where, after the hydrophilic groups were grafted onto bisphenol-A (E44) and a phase transformation process occurred, the active emulsion served as multiple crosslinkers to construct physical and chemical interactions with soy protein molecules to form a stable crosslinking network. Afterwards, the effects of the neutralisers were studied, namely triethylamine (TEA) and N,N-dimethyl ethanolamine (DMEA). The resulting modified soy protein-based adhesive exhibited a more compact fractured morphology and improved thermal stability and water resistance when compared with a simple soy protein (SP) adhesive. The use of (TEA) produced an adhesive with an increment of 20% of wet shear strength compared to one produced using (DMEA), and this adhesive reached 1.14 MPa, a 192.5% increase when compared with a simple soy protein-based adhesive. The final properties meet the requirement of PLW for interior use, which is ≥0.7 MPa [79].

A report was recently published in the European Polymer Journal by Zeng et al. (2021) [70] where a crosslinker (BHTA) was prepared through the reaction between 10 g of 1,6-hexadiol glycidyl ether (BEPH) and 1 g of triethylenetetramine (TETA) at 50 °C with constant stirring for 30 min.

This crosslinker was then added to 15 g of denatured soy protein and mixed with 85 g of deionised water (the denaturation occurred with the addition of 0.6 g of borax and 0.36 g of sodium sulphite). The prepared adhesive was used to obtain a PLW sample, which was later tested with other adhesive formulation samples. The results showed an increase of 151% (2.79 MPa) and 409% (1.12 MPa) in dry and wet strength, respectively, compared with a simple SPI-based adhesive. Both the dry and wet strength results exceeded the requirements to meet the standard of type II plywood (>0.7 MPa according to GB/T17657-2013 [82]) and this provides an essential and relevant route to produce SPI-based adhesives with good water resistance.

Other promising results show that molecular recombination can produce a cleaner final adhesive while using lower amounts of crosslinking agents. A report described the introduction of bromelain to degrade soy protein into small peptide chains. Bromelain was added at different amounts, from 0.05 to 0.4%, and the molecular weight of the protein decreased to below 25 KDa, with the viscosity of the resultant adhesive decreasing also from 147,000 to 18,056 mPa.s. When a crosslinking agent, triglycidylamine (TGAm), was added, viscosity was found to be as low as 1125 mPa.s. These two steps generated a uniform and stable crosslinked network structure that improved the performance and bond stability of the adhesive. The results showed that while the viscosity decreased with each addition of bromelain, the wet shear strength of PLW samples made with the produced adhesives peaked when the bromelain added was 0.1 g. This sample still presented a respectable 95% decrease in viscosity while producing a wet shear strength of 1.11 MPa, an increase of 76.2% compared with a simple protein isolate adhesive. In addition, compared with other crosslinked modified soy protein adhesives, these tests showed similar results while needing 50% less crosslinking agent [83].

Polyacrylate emulsions have been employed to synthesise soy protein–polyacrylate emulsions that could be used as adhesives for WBPs. The results of tests performed show that the viscosity decreases with the increase in polyacrylate emulsion, facilitating the wetting and penetration of wood. The introduction of neutralised poly(acrylic acid) is a possible reason for the increase in shear strength results when tested in PLW samples.

It was also observed that the addition of 3% of MDI to the adhesive, when tested for shear strength, produces results above 0.7 MPa [84].

Other epoxy-based crosslinker formulations for use with SPI are described by Mousavi et al. (2021) [85], in which a combination of bisphenol A diglycidyl ether (DGEBA) and PEI was mixed with SPI in order to produce an adhesive that could create crosslinking networks at room temperature. The studied adhesive formulation required 233 g of deionised water, 37.5 g of SPI, 10 g of DGEBA, and 5 g of a 50 wt% aqueous solution of PEI. The adhesive preparation was performed at room temperature. The PLW samples were prepared at room temperature, pressed at 1.0 MPa and room temperature for 2 h. The samples were then stored at standard conditions for six days before being subjected to a slew of tests, such as a cycle boil shear test, dry shear test, and two-cycle boil test according to the American National Standard for Hardwood and Decorative Plywood (ANSI/HPVA HP-1-2020) [86]. The results indicated that the samples started to fail in the two-cycle boil and cycle boil shear tests for weight rations of SPI/PEI 20 or above. All the other formulations tested presented appealing results, mainly above the requirements, with surprisingly good results for an SPI/PEI ratio of 15, achieving a local maximum for both the dry shear test and the cycle boil shear test, which were well above the 0.7 MPa required for type II plywood [82]. The same study identified that formulations with DGEBA/(SPI+PEI) weight ratios of 1.15 presented the highest results for the dry shear strength and cycle boil shear strength, 1.69 MPa and 1.31 MPa, respectively. Choosing this formulation, however, would imply high consumption of DGEBA that could be avoided, since the results for formulations with weight ratios of 0.55 and 0.40 also provided good results for the dry shear test, 1.60 and 1.40 MPa, respectively, and cycle boil shear tests, 1.19 and 1.22 MPa, respectively, which makes these formulations with lower weight rations more attractive in research, and possibly commercially.

#### 5.3.2. Cottonseed

Although soy proteins are currently the most well-known and researched type of feedstock being used commercially as a “green” wood adhesive, studies [81] show that cottonseed protein isolate has the potential to produce adhesives with higher adhesive strength and hot water resistance when compared directly with the properties of adhesives based on soy protein isolates [87].

Cottonseed protein is a promising feedstock source, functioning as well as an alternative source in adhesive production since not only does its use not compete with any type of food industry, unlike soy protein, but its isolate has been shown to produce adhesives with better performance than the SPI-based ones, even though there is still room for improvement [71]. Cottonseed protein is also a potential alternative to formaldehyde-based adhesives as a renewable feedstock. As with other protein-based adhesives, unmodified cottonseed protein isolate needs improved water resistance to replace petrochemical-based adhesives [71].

It has been shown that cottonseed protein isolate-based adhesives lead to better properties in the final product when compared with cottonseed meal. However, the protein isolate extraction and isolation processes are costly. In order to alleviate this cost, studies have been performed with water-washed cottonseed meal-based adhesives, which are cheaper to produce and therefore can be more efficiently used in industrial production. The water-washed cottonseed meal has a lower concentration of proteins than the cottonseed protein isolate. In order to use it efficiently in adhesive production, the limits on the amount of protein needed in the adhesive mix to still generate an adhesive with good properties have to be regulated since the protein content has a greater impact on thermal properties and bonding connections than, for instance, press temperature [88]. It was concluded by Pradyawong et al. (2018) [88] that a protein content of 65 to 70% of water-washed cottonseed meal was needed for performance comparable with that of a cottonseed protein isolate. In the same study, the authors also developed models to estimate the wet, dry, and soaked strengths in MPa of plywood samples. The developed models’ variables were the temperature, which could be from 100 to 170 °C, and the protein concentration, from 34.9% to 94.8% in wet samples and 70.6% for both dry and soaked samples.

The performance of a protein in an adhesive formulation likely depends on the reaction of protein with the wood surface and the formation of crosslinked networks among the denatured protein molecules during the heat bonding process.

A possible cause for the denaturation of proteins from cottonseed and soy is the interactions between arginine found in proteins (among other cationic moieties) and carboxylate anions encountered in the additives butyric, glutamic, and aspartic acids. These interactions are believed to facilitate protein denaturation and destabilisation and improve bonding strength [81]. Cottonseed proteins possess a more significant amount of arginine, between 11 and 12%, than those of soy, usually between 7 and 8% [81], which could be one of the reasons that, when comparing them, the cottonseed protein-based adhesives perform better than soy protein-based ones. Arginine carries a net cationic charge at most pH values (up to pH 12), so the added carboxylate anions may interact with it, and with other cationic moieties present, to form anion–protein bridge interactions. Arginine also decreases heat-induced protein aggregation, making the protein denaturation occur more quickly at temperatures above 80 °C [81].

Some modifications of protein formulations have been shown to affect the adhesive properties of soy and cottonseed protein isolate. A method tested to improve the adhesion properties of the final product was the addition of protein modifiers to the formulation. Some of these additives can be more environmentally friendly, such as amino and fatty acids [2]. Research into modifiers to be used in the adhesive formulation to improve its properties has already shown promising results, with one such example being the use of phosphoric acid, H_3_PO_4_ [71]. Further developments in this process were studied by Li et al. (2021) [89], where both CaCl_2_ and Ca(NO_3_)_2_ were mixed with phosphoric acid, with the report indicating that better test results were achieved when the molar concentrations, in mM, of both H_3_PO_4_/CaCl_2_ and H_3_PO_4_/ Ca(NO_3_)_2_ were 40/10 and 40/20. This same study optimised the reagent proportions found in the adhesive formulation amounts of H_3_PO_4_, CaCl_2_, and Ca(NO_3_)_2_ since lower amounts for each reagent may lower the proteins’ denaturation whilst at the same time lowering the crosslinking. Higher amounts of H_3_PO_4_ may cause excessive CPI denaturation due to higher acidity, whilst higher amounts of CaCl_2_ and Ca(NO_3_)_2_ may lead to excessive CPI denaturation, in both mentioned cases, lowering the adhesive properties of the proteins.

The added amino acids whose results showed significant improvements were mostly smaller molecules that could carry anionic charges, such as aspartic, glutamic, or acetic acid [81]. These modifiers also showed relevant positive effects regarding the adhesive’s hot water resistance when used with cottonseed protein isolate, whilst, in the same circumstances, modified SPI-based adhesives’ hot water resistance was markedly worse [81].

In another study [90], guanidine hydrochloride (GdnHCl) and SDS were tested as denaturing agents for water-washed cottonseed meal to improve the bonding capability and viscosity of the resulting adhesive for use in small wood item bonding. The results show that the highest strength was found in an adhesive with a formulation of 30% of water-washed cottonseed meal and 19.1% of GdnHCl, in solid content. The tests performed with SDS added a stronger denaturing agent than GdnHCl and had lower overall strength test results, even though, for the lowest limit of press time tested, 60 min, the results were comparable. This study also revealed that the longer the press times that the adhesives with GdnHCl added were subjected to, the higher the tensile shear strength results that were obtained [90].

Tung oil was tested as an additive for cottonseed meal and protein isolate-based adhesives to improve water resistance and adhesion strength. The results showed that the addition of tung oil to the adhesive mix increased the resulting adhesive’s strength by 21.1% for all cottonseed protein isolate-based adhesives and 19.9% for water-washed cottonseed meal-based adhesive formulations when compared with the same adhesive formulations without tung oil. The adhesives with tung oil added also had better water resistance, with improvements of 46.6 and 41.3% for water-washed cottonseed meal and cottonseed protein isolate, respectively [91].

## 6. Life Cycle Assessment of Bio-Based Adhesives

A significant parameter to consider when developing a new product or manufacturing process is their resulting ecological impact. It is necessary to compare the current industry standard, such as the use of petrochemical-based adhesives in the manufacturing process of PB, with new adhesives developed by an alternative, more environmentally friendly route. A helpful tool employed to analyse the carbon footprint is realising a life cycle assessment (LCA) of the adhesive production process. As an example of the importance of developing bio-based adhesives, a number of LCA studies have been performed around the world, such as the report by Balasbaneh et al. (2021) [92] regarding engineered wood-based construction materials, which denounces adhesives as the principal factor in ozone layer depletion emissions, as well as a significant factor in the global warming potential (GWP), in land use potential, and in fossil depletion potential. Murphy et al. (2015) [93] tackled the LCAs resulting from the exploration of the wood processing industry in Ireland. In their conclusions for the manufacture of MDF and OSB boards, they found synthetic, non-renewable sourced adhesives to be responsible for 62% and 35% of the GHG emissions, respectively. A similar conclusion regarding the use of synthetic petrochemical-based adhesives for the manufacture of fibreboards was reached by Gonzalez-García et al. (2009) [94]. This study considered the use of PF resin in the manufacturing process, finding its usage responsible for approximately 33% of GWP, with a human toxicity potential above 91%, as well as being significantly responsible for terrestrial and marine aquatic ecotoxicity potentials, among other studied parameters. It was therefore concluded that the development of bio-based adhesives for use in the manufacturing process should be a priority to reduce the consequences of maintaining an industry as large as the wood-based panel manufacture industry that is dependent on petrochemical-sourced adhesives.

An LCA study performed on the principal wood processing industries in Portugal [95] has found that, for the manufacture of PB, adhesive resin (UF and MF) contributes to approximately 62% of the environmental impact caused during the products’ manufacture stage, with the authors suggesting the use of non-formaldehyde-based resins for the manufacture process.

Some LCAs conducted throughout the world showcase some differences in the manufacturing processes of PB, with each region presenting different challenges and “hotspots” that can be improved through innovation. An example of such a case is the use of UF resins as the primary adhesive in manufacturing processes, which has repeatedly been reported as a disproportional, and sometimes more significant, contributor to the environmental impact of the final product [96,97,98]. According to published data, the average amount of UF resin used in the production line by cubic metre of manufactured PB varies by region, including 71.7 kg in Brazil [96], 67.9 kg in Spain [96], and 93 kg in Pakistan [97].

An example of different priority hotspots for the PB manufacturing process found for an LCA performed comparing the PB manufacturing lines in Spain and Brazil [96] was the energy used by the respective production lines and the type of adhesive used, and the primary source of raw material. While the Brazilian production lines obtained their energy mainly from the national power grid, which is still primarily fossil-fuel-based and has a more considerable environmental impact, the Spanish manufacturing lines already implement cogeneration units that can use wood waste from factories as a source of fuel. In the Spanish example, the energy produced from cogeneration corresponded to approximately 60% of the total energy requirement for the manufacturing process, which led the study’s authors to propose the application of a similar method for the Brazilian production lines, which, by their estimations, could be responsible for 66% of the energy requirements per cubic metre of PB produced.

In the report by Arias et al. (2020) [20], the bio-based adhesives tested provided disappointing results in their LC compared to currently used formaldehyde-based adhesives. The authors suggested that more sustainable crosslinkers should be used in the formulations, and improved adhesive-related properties should be achieved to consider this type of adhesive as a viable “greener” alternative to the currently used ones.

## 7. Conclusions and Future Trends

The development of sustainable and environmentally friendly adhesives in the WBP industry has become a focus of research to respond to new regulations and problems found in the use of conventional adhesive systems. For instance, replacing formaldehyde-based adhesives is a critical factor in the industry’s sustainable growth, leading to the more widespread adoption of artificially manufactured WBPs in applications such as interior furniture decoration and structural engineering.

The adoption of pMDI as the primary adhesive type in the WBP industry still poses issues due to the petrochemical origin of most of the commercially used isocyanates. Therefore, the transition to greener adhesives is mainly focused on developing bio-based polymers, obtained primarily from several sources, namely proteins, tannins, or lignin, among others. These sources of adhesives present several advantages, such as the valorisation of residues/waste from other industrial processes, which makes them economically attractive. On the other hand, the cost of bio-based adhesives derived from the typically lower reactivities of the bio-based raw materials and associated randomness are significant hurdles that need to be overcome. Some progress has been made, with promising leads regarding the manufacture of panels for furniture and other indoor uses and other structural functions in a dry environment. Nonetheless, water resistance and biodegradation still seem to represent significant problems to be satisfactorily solved, permitting the use of the produced panels in outdoor conditions, which are much harsher and may lead to much higher mechanical and physical degradation.

Most of the progress in this area of development has been made on a lab scale, with the transition to an industrial scale still proving difficult, with issues ranging from technical to economical. Nevertheless, some key issues have been identified, such as water resistance and the need for more economical adhesive formulations, and are currently being investigated, with some promising results.

The imposition of stricter regulations and increased social pressure will further incentivise these newer technologies, leading to the greater allocation of resources dedicated to this research area. This new panorama should mitigate typical issues found in the development of greener, more sustainable manufacturing lines—for example, the biomass availability for feedstocks—and it should also accelerate the development and implementation of novel bio-adhesive technologies. The physical and mechanical performance of the new bio-adhesives and their flexibility in terms of varied applications are critical aspects for their penetration of the market.

There is still urgent R&D to be done on bio-based adhesives, targeting more efficient processes and greater availability to fulfil the specific and sometimes niche requirements of the WBP market. However, some glimpses of a better and greener future can be already found in the current published work from around the world, some of which is mentioned in this state of the art, as well as from the emerging novel trends in the industry, often driven by new restrictions and regulations, towards the replacement of dangerous, volatile components in adhesive formulations.

## Figures and Tables

**Table 1:** Types of PBs manufactured in Europe according to EN 312 standard, as shown in [11].

Type	PB Application	Standard
P1	General-purpose boards for use in dry conditions	EN 312:2010
P2	Boards for interior fitments (including furniture) for use in dry conditions	EN 312:2010
P3	Non-load-bearing boards for use in humid conditions	EN 312:2010
P4	Load-bearing boards for use in dry conditions	EN 312:2010
P5	Load-bearing boards for use in dry conditions	EN 312:2010
P6	Heavy-duty load-bearing boards for use in dry conditions	EN 312:2010
P7	Heavy-duty load-bearing boards for use in humid conditions	EN 312:2010

**Table 2:** Grades of OSB manufactured in Europe, according to EN 300 standard [3].

Grade	Use
OSB/1	General-purpose boards and boards for interior fitments (including furniture) for use in dry conditions
OSB/2	Load-bearing boards for use in dry conditions
OSB/3	Load-bearing boards for use in humid conditions
OSB/4	Heavy-duty load-bearing boards for use in humid conditions

**Table 3:** Types of MDF manufactured in Europe according to EN 622-5 standard, as shown in [17].

Type	Board Application	Standard
MDF	General-purpose boards for use in dry conditions	EN 622-5
MDF.H	General-purpose boards for use in humid conditions	EN 622-5
MDF.LA	Load-bearing boards for use in dry conditions	EN 622-5
MDF.HLS	Load-bearing boards for use in humid conditions	EN 622-5
L-MDF	Light-MDF boards for use in dry conditions	EN 622-5
L-MDF.H	Light-MDF boards for use in humid conditions	EN 622-5
UL1-MDF	Ultra-light-MDF boards for use in dry conditions	EN 622-5
UL2-MDF	Ultra-light-MDF boards for use in dry conditions	EN 622-5
MDF-RWH	Boards for use in rigid underlays in roofs and walls	EN 622-5

**Table 4:** Tests performed on adhesives.

**ASTM D1183-03**	Standard Practices for Resistance of Adhesives to Cyclic Laboratory Aging Conditions [32]
**ASTM D1875-03**	Standard Test Method for Density of Adhesives in Fluid Form [33]
**ASTM D1151-00**	Standard Practice for Effect of Moisture and Temperature on Adhesive Bonds [34]
**ASTM D1828-01**	Standard Practice for Atmospheric Exposure of Adhesive-Bonded Joints and Structures [35]
**ASTM D903-98**	Standard Test Method for Peel or Stripping Strength of Adhesive Bonds [36]
**ASTM D1876-08**	Standard Test Method for Peel Resistance of Adhesives (T-Peel Test) [37]
**ASTM D905-08**	Standard Test Method for Strength Properties of Adhesive Bonds in Shear by Compression Loading [38]
**ASTM D1337-10**	Standard Practice for Storage Life of Adhesives by Viscosity and Bond Strength [39]
**ASTM D1084-16**	Standard Test Methods for Viscosity of Adhesives [40]

## Data Availability

Not applicable.
